# Long Noncoding RNA/Circular RNA-miRNA-mRNA Axes in Ischemia-Reperfusion Injury

**DOI:** 10.1155/2020/8838524

**Published:** 2020-11-25

**Authors:** Chengwu Gong, Xueliang Zhou, Songqing Lai, Lijun Wang, Jichun Liu

**Affiliations:** ^1^Department of Cardiothoracic Surgery, Second Affiliated Hospital, Nanchang University, Nanchang, Jiangxi 330006, China; ^2^Department of Cardiothoracic Surgery, First Affiliated Hospital, Nanchang University, Nanchang, Jiangxi 330006, China

## Abstract

Ischemia-reperfusion injury (IRI) elicits tissue injury involved in a wide range of pathologies. Multiple studies have demonstrated that noncoding RNAs (ncRNAs), including long noncoding RNAs (lncRNAs), circular RNAs (circRNAs), and microRNAs (miRNAs), participate in the pathological development of IRI, and they may act as biomarkers, therapeutic targets, or prognostic indicators. Nonetheless, the specific molecular mechanisms of ncRNAs in IRI have not been completely elucidated. Regulatory networks among lncRNAs/circRNAs, miRNAs, and mRNAs have been the focus of attention in recent years. Studies on the underlying molecular mechanisms have contributed to the discovery of therapeutic targets or strategies in IRI. In this review, we comprehensively summarize the current research on the lncRNA/circRNA-miRNA-mRNA axes and highlight the important role of these axes in IRI.

## 1. Introduction

Ischemia-reperfusion injury (IRI) occurs after an initial restriction of blood supply to an organ followed by restoration of perfusion [1]. The mechanisms contributing to the pathogenesis of IRI include oxidative/nitrosative stress, mitochondrial dysfunction, calcium overload, inflammation, and activation of apoptotic and autophagic pathways, among other mechanisms [2]. Studies have reported single-target interventions for these pathogeneses of IRI. Nitric oxide (NO) reduces mitochondrial damage and reactive oxygen species (ROS) derived from reperfusion by mimicking the protective effect of kinase pathways that decrease apoptosis and tissue damage. However, it has been difficult to determine the optimal NO dose, and excess NO levels have been determined to be harmful [3]. To reduce the calcium overload, the inhibition of proteins, of which sustained activation causes excessive cation influx, is believed to have a protective effect in ischemia models [4]. When preventative strategies against IRI cannot be used, suppression of the inflammatory response is beneficial for IRI. However, the inflammatory pathways are so complex that blocking any medium in the system may not provide definitive and effective treatment [5]. Compared with single-target interventions, multitarget interventions may have better efficacy in the treatment of IRI.

For decades, research has focused on the 2% of the human genome that codes for proteins [6]. In recent years, researchers have found that the remaining 98% of the genome that was once considered as nonfunctional “junk” includes noncoding RNAs (ncRNAs) that play important roles in a wide range of biological processes such as growth, development, and organ function. Furthermore, ncRNAs have been found to function in all kinds of human diseases and conditions, including IRI [7–9]. MicroRNAs (miRNAs) are a family of ncDNAs comprising 21–25 nucleotides and are the most commonly researched class of ncRNAs. miRNAs play essential regulatory roles in the expression of proteins by binding specific target mRNAs for cleavage or translational repression [10]. Long noncoding RNAs (lncRNAs), the class of ncRNA making up the largest portion of the mammalian ncRNAs, are a heterogeneous group of ncRNAs more than 200 nucleotides long that regulate gene expression through a diverse range of mechanisms [11]. Circular RNAs (circRNAs), characterized by their covalently closed-loop structures without 5′ caps and 3′ poly tails, comprise a large class of ncRNAs that are produced by a noncanonical splicing event called back-splicing [12]. Recent studies have also revealed a role of lncRNAs/circRNAs as competing endogenous RNAs (ceRNA) that sponge specific miRNAs to indirectly regulate the expression of many genes. Increasing evidence has identified the abnormal expression of ncRNAs in IRI of multiple organs, especially the heart, brain, liver, and kidney [13–16]. Furthermore, several studies have identified lncRNAs/circRNAs that function as ceRNAs in regulating the expression of many genes as vital to the development and progression of IRI, which may provide multitarget interventions for the treatment of IRI.

**Figure 1:**
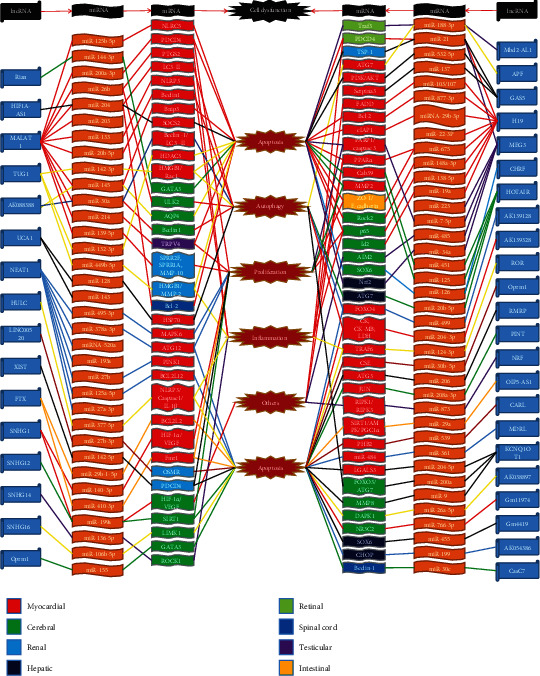
lncRNA-miRNA-mRNA axes in ischaemia/reperfusion injury. lncRNA-miRNA-mRNA axes regulating the pathogenesis of ischemia/reperfusion injury are shown, which are associated with cell apoptosis, autophagy, and proliferation, as well as inflammation and others. Colors in the boxes represent different organs. Colors in lines mean that different lncRNAs target their corresponding miRNAs and mRNAs. Abbreviations: lncRNA: long noncoding RNA; miRNAs: microRNAs; Rian: RNA imprinted and accumulated in nucleus; HIF1A-AS1: hypoxia inducible factor 1*α*-antisense RNA 1; MALAT1: metastasis-associated lung adenocarcinoma transcript 1; TUG1: taurine-upregulated gene 1; Mbd2: methyl-CpG-binding domain protein 2; APF: autophagy-promoting factor; GAS5: growth arrest specific 5; MEG3: maternally expressed gene 3; CHRF: cardiac hypertrophy-related factor; HOTAIR: HOX transcript antisense RNA; NLRC5: nucleotide-binding and oligomerization domain-like receptor C5; AKT: protein kinase B; PDCD4: programmed cell death 4; PTGS2: prostaglandin-endoperoxide synthase 2; NLRP3: Nod-like receptor protein-3; BNIP3: Bcl2 19 kDa protein-interacting protein 3; SOCS2: suppressor of cytokine signaling 2; HDAC3: histone deacetylase 3; HMGB1: high-mobility group box 1; Rac1: Ras-related C3 botulinum toxin substrate 1; GATA3: GATA-binding protein 3; ULK2: Unc-51-like kinase 2; AQP4: aquaporin 4; TRPV4: transient receptor potential vanilloid 4; SPRR2F: small proline-rich protein 2F; SPRR1A: small proline-rich protein 1A; MMP-10: matrix metalloproteinase-10; HMGB1: high-mobility group box 1; MMP-2/8: matrix metalloproteinase-2/8; Bcl2: B-cell lymphoma-2; Traf3: tumor necrosis factor (TNF) receptor-associated factor 3; PDCD4: programmed cell death 4; TSP-1: thrombospondin 1; PI3K: phosphatidylinositol 3 kinase; FADD: Fas-associated protein with death domain; cIAP1: cellular inhibitor of apoptosis protein 1; PARP1: poly(ADP-ribose) polymerase 1; PPAR*α*: peroxisome proliferator-activated receptor *α*; Cab39: calcium-binding protein 39; ZO-1: zonula occludens 1; Rock2: rho-associated protein kinase 2; Id2: inhibitor of DNA binding/differentiation 2; AIM2: absent in melanoma 2; SOX6: sex-determining region Y box 6; Nrf2: nuclear factor erythroid 2-related factor; UCA1: urothelial carcinoma-associated 1; NEAT1: nuclear paraspeckle assembly transcript 1; HULC: highly upregulated in liver cancer; XIST: X chromosome inactivation; FTX: five prime to Xist; SNHG1/12/14/16: small nucleolar RNA host gene 1/12/14/16; RMRP: mitochondrial RNA-processing endoribonuclease; PINT: p53-induced transcript; NRF: necrosis-related factor; OIP5-AS1: Opa-interacting protein 5-antisense transcript 1; CARL: cardiac apoptosis-related lncRNA; MDRL: mitochondrial dynamic-related lncRNA; KCNQ1OT1: KCNQ1 opposite strand/antisense transcript 1; CasC7: cancer susceptibility candidate 7; HSP70: heat shock protein70; MAPK6: mitogen-activated protein kinase 6; ATG3/7/12: autophagy-related gene 3/7/12; PINK1: PTEN-induced putative kinase 1; Bcl2l2/12: B-cell lymphoma-2-like 2/12; IL-1*β*: interleukin-1*β*; HIF-1*α*: hypoxia inducible factor-1*α*; VEGF: vascular endothelial growth factor; Fmr1: fragile X mental retardation 1; OSMR: oncostatin M receptor *β*; SIRT1: sirtuin 1; LIMK1: the LIM motif-containing protein kinase family-contained LIM kinase 1; ROCK1: rho-associated coiled-coil-containing protein kinase 1; FOXO3/4: forkhead box O3/4; CK: creatine kinase; CM-MB: creatine kinase MB form; LDH: lactate dehydrogenase; TRAF6: TNF receptor-associated factor 6; CSE: cystathionine-*γ*-lyase; RIPK 1/3: receptor-interacting serine/threonine-protein kinase 1/3; AMPK: adenosine monophosphate-activated protein kinase; PGC1*α*: peroxlsome proliferator-activated receptor-*γ* coactlvator-1*α*; PHB2: prohibitin 2; LGALS3: galectin-3; DAPK1: death-associated protein kinase 1; NR3C2: nuclear receptor subfamily 3 group C member 2; CHOP: C/EBP homologous protein.

**Figure 2:**
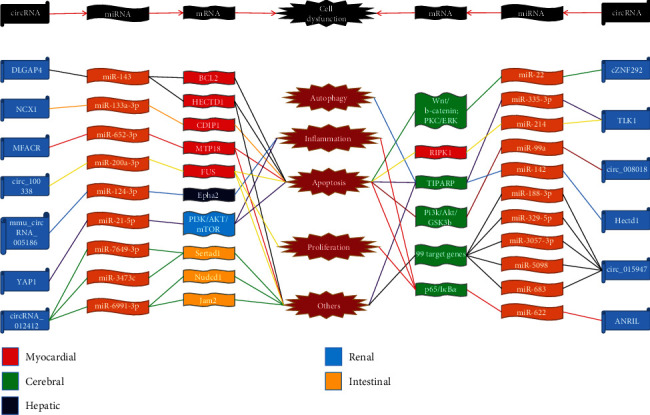
circRNA-miRNA-mRNA axes in ischaemia/reperfusion injury. circRNA-miRNA-mRNA axes regulating the pathogenesis of ischemia/reperfusion injury are shown, which is associated with cell apoptosis, autophagy, and proliferation, as well as inflammation and others. Colors in the boxes represent different organs. Colors in lines mean that different circRNAs target their corresponding miRNAs and mRNAs. Abbreviations: circRNA: circular RNA; miRNAs: microRNAs; NCX1: sodium/calcium exchanger 1; MFACR: mitochondrial fission and apoptosis-related circRNA; ANRIL: antisense noncoding RNA in the INK4A locus; YAP1: yes-associated protein 1; TLK1: serine/threonine-protein kinase tousled-like 1; Bcl2: B-cell lymphoma protein 2; HECTD1: HECT domain E3 ubiquitin protein ligase 1; CDIP1: cell death-inducing protein; MTP18: mitochondrial protein 18 kDa; RIPK1: receptor-interacting serine/threonine-protein kinase 1; Epha2: ephrin type-A receptor 2; PI3K: phosphatidylinositol 3-kinase; AKT: protein kinase B; mTOR: mammalian target of rapamycin; Sertad1: SERTA domain-containing protein 1; Nudcd1: NudC domain-containing protein 1; Jam2: junctional adhesion molecule B; PKC: protein kinase C; ERK: mitogen-activated protein kinase; TIPARP: TCDD inducible poly(ADP-ribose) polymerase; GSK3B: glycogen synthase kinase 3 beta.

In this review, we provide an overview of the roles of the lncRNA/circRNA-miRNA-mRNA axis as potential biomarkers and therapeutic targets for the detection and treatment of IRI in different organs, including the heart, brain, liver, and kidney **(**Figures 1 and 2**)** and as mediators and effectors of organ protection. In addition, we discuss prospective tactics for targeting ncRNAs as potential novel therapies for IRI to reduce tissue injury of important organs.

## 2. lncRNA/circRNA-miRNA-mRNA Axis in IRI

Tissue injury elicited by ischemia and reperfusion (I/R) occurs in a wide range of pathologies, especially in myocardial infarction (MI), ischemic stroke of the brain, acute kidney injury (AKI), intestinal ischemia, retinal vascular occlusion, and organ transplantation [1]. Previous studies have shown that ncRNAs play an important role in I/R. Multiple pathological processes that contribute to I/R are associated with cell dysfunction, including apoptosis and necrosis, or autophagy dysfunction, cell proliferation, and sterile inflammation [1, 2, 17].

### 2.1. Heart

Cell death is a cardinal contributor to most cardiac diseases such as MI, IRI, and heart failure [18]. The high morbidity and mortality of cardiac diseases is mainly caused by myocardial cell death due to I/R [19]. See Table 1 for a summary of the studies of the lncRNA-miRNA-mRNA axes in myocardial IRI.

#### 2.1.1. lncRNA/circRNA-miRNA-mRNA Axis Regulates Apoptosis of Cardiomyocytes in IRI

Apoptosis is the major form of programmed cell death. Accumulating evidence has demonstrated that the lncRNA/circRNA-miRNA-mRNA axis plays an important role in IRI by mediating cell apoptosis. lncRNA metastasis-associated lung adenocarcinoma transcript 1 (MALAT1) was reported to promote cardiomyocyte apoptosis in an IRI-induced MI mouse model via PDCD4 (Programmed cell death 4) upregulation by sponging miR-200a-3p [20]. Another study suggested that MALAT1 may function as a ceRNA to upregulate NLRC5 (nucleotide-binding and oligomerization domain-like receptor C5) by binding to miR-125b-5p in an IRI-induced acute myocardial infarction (AMI) mouse model, leading to the apoptosis of myocardial cells [21]. Another report showed that the cardiac protective effect of fentanyl was abrogated by MALAT1 through its negative regulation of the miR-145/Bnip3 (Bcl2 19 kDa Protein-Interacting Protein 3) pathway [22].

The lncRNA H19 is transcribed from the imprinted H19-insulin growth factor 2 locus [23]. The exact functions of H19 in cancer have been controversial because it has been identified not only as an oncogene but also a tumor suppressor. Similarly, H19 seems to have a contradictory effect in IRI. Some studies showed that the overexpression of H19 reduced cell apoptosis and alleviated myocardial IRI of mice and cardiomyocyte injury induced by H_2_O_2_ or hypoxia-reoxygenation (H/R) through interacting with different miRNAs and mRNAs [24–26]. Conversely, Luo et al. [27] showed that knockdown of H19 promoted cell viability, inhibited cell apoptosis, reduced inflammatory cytokines, suppressed oxidative stress, and decreased infarct size in a myocardial I/R mouse model through the miR-675/PPAR*α* (Peroxisome proliferator-activated receptor *α*) pathway. Further research is needed to clarify the definitive mechanism and function of H19 in IRI of the heart.

The lncRNA nuclear-enriched abundant transcript 1 (NEAT1), transcribed from a common promoter by RNA polymerase II, is commonly expressed in mammalian cells and acts as a scaffold for the nucleus [28–30]. Multiple studies that investigated the effects of NEAT1 in myocardial IRI demonstrated that NEAT1 was abnormally upregulated *in vitro* and *in vivo* [31–34]. NEAT1 was also significantly upregulated in peripheral blood of patients with unstable angina and patients with ischemic cardiomyopathy/MI in comparison with healthy controls [34]. Furthermore, the overexpression of NEAT1 promoted the apoptosis of cardiomyocytes and enhanced myocardial IRI via different axes, such as the NEAT-miR-495-3p-MAPK6 and NEAT-miR-27b-PINK1 axes [31, 35]. However, Yan et al. reported the opposite results and found that NEAT1 was downregulated in cardiomyocytes following IRI *in vivo* and hydrogen peroxide treatment *in vitro* and acted as a miRNA sponge to target miR-125a-5p, leading to the upregulation of Bcl12l12 (B-cell lymphoma-12-like 12) and the inhibition of cardiomyocyte apoptosis [36].

Previous studies showed that suppression of the lncRNA HOX transcript antisense RNA (HOTAIR) exasperated cell viability and migration potential and increased apoptosis induced by oxidative stress in H9C2 cells, which may be partly attributed to the HOTAIR/miR-125/MMP-2 (Matrix metalloproteinase-2) axis [37]. Correspondingly, HOTAIR prevented oxidative stress and cardiac myocyte apoptosis in myocardial IRI, which involves AMPK*α* activation via the EZH2/miR-451/Cab39 axis [38]. Yu and Chen [39] speculated that circulating HOTAIR/miR-126 may be a potential biomarker and risk factor predictor for myocardial IRI.

Multiple studies have investigated the effects of other lncRNA-miRNA-mRNA axes in myocardial IRI on myocardial cell apoptosis. Enhanced expression of the lncRNA five prime to XIST (FTX) curbed cardiomyocyte apoptosis via targeting the miR-29b-1-5p/Bcl2l2 and miR-410-3p/Fmr1 axes [40, 41]. The mitochondrial dynamic-related lncRNA (MDRL) reduces mitochondrial fission and apoptosis upon I/R by downregulating miR-361, which inhibits the progression of miR-484 by binding directly to the primary transcript of miR-484 and precludes Drosha from processing it into pre-miR-484 [42]. The cardiac apoptosis-related lncRNA (CARL) functions as a ceRNA to sponge miR-539 and regulate prohibitin 2 (PHB2) expression, mitochondrial fission, and apoptosis in myocardial IRI mice and mouse primary cardiomyocytes under anoxia [43]. Moreover, silencing of the lncRNA growth arrest specific 5 (GAS5) promoted the activation of the PI3K/AKT-mediated apoptosis pathway, potentially by sponging miR-532-5p, in myocardial IRI rats and H9C2 cells under H/R treatment [44]. Liu et al. [45] speculated that GAS5 also aggravates myocardial IRI by regulating miR-137/serpina3. Downregulation of hypoxia inducible factor 1*α*-antisense RNA 1 (HIF1A-AS1) and upregulation of miR-204 inhibited myocardial cell apoptosis to mitigate ventricular remodeling and ameliorate cardiac function in a myocardial IRI mouse model via regulating SOCS2 (Suppressor of cytokine signaling 2) [46]. Downregulation of the lncRNA KCNQ1 opposite strand/antisense transcript 1 (KCNQ1OT1) resulted in the reduction of the apoptosis rate of myocardial tissues and the alleviation of myocardial IRI in mouse models through the miR-204-5p/LGALS3 axis [47]. Downregulation of the lncRNA maternally expressed gene 3 (MEG3) protected myocardial cells against I/R-induced apoptosis through the miR-7-5p/PARP1 pathway [48]. Yu et al. [49] speculated that the MEG3/miR-223 axis may play vital roles in the prediction and biological labeling of myocardial IRI. Furthermore, the lncRNA p53-induced transcript (PINT) activated the MAPK pathway to facilitate myocardial IRI and apoptosis and promoted AMI by regulating miR-208a-3p/JUN [50]. Recently, the novel lncRNA mitochondrial RNA-processing endoribonuclease (RMRP) was found to inhibit the viability, migration, and invasion abilities of H9C2 cells with hypoxia treatment; RMRP may aggravate myocardial IRI by targeting miR-206, leading to ATG3 upregulation [51]. The lncRNA taurine-upregulated gene 1 (TUG1) plays critical roles in the pathogenic development of AMI through regulating the miR-132-3p/HDAC3 and miR-142-3p/HMGB1 (High-mobility group box 1)/Rac1 axes. TUG1 significantly inhibited cell viability, promoted cell apoptosis, promoted cell autophagy, and increased the production of ROS in cardiomyocytes treated with H_2_O_2_ and aggravated I/R-induced AMI in a mouse model [52, 53]. Yu et al. [54] confirmed that the lncRNA urothelial carcinoma-associated 1 (UCA1) interferes with miR-143 expression to modulate cardiomyocyte apoptosis in myocardial IRI. The lncRNA small nucleolar RNA host gene 1 (SNHG1) was found to promote the proliferation and enhance the function of human umbilical vein endothelial cells by activating the HIF-1*α*/VEGF signaling pathway though miR-140-3p [55]. In addition, ROR and HULC both modulated myocardial IRI in rat models and H/R-induced inflammation and cell apoptosis through the miR-124-3p/TRAF6 and miR-377-5p/NLRP3 (Nod-like receptor protein-3)/Caspase-1/IL-1*β* axes, respectively [56, 57]. Hu et al. [58] showed that the lncRNA Oprm1 alleviated myocardial IRI to preserve cardiac function, increased cystathionine-*γ*-lyase activity, and inhibited cell apoptosis through miR-30b-5p, in which activation of the PI3K/Akt pathway and inhibition of HIF-1*α* activity are involved. Furthermore, the lncRNA Opa-interacting protein 5-antisense transcript 1 (OIP5-AS1) alleviated oxygen-glucose deprivation and reperfusion- (OGD/R-) induced cell apoptosis, oxidative stress, and mitochondrial membrane depolarization in H9c2 cells; OIP5-AS1 prevented OGD/R injury via regulation of miR-29a and the SIRT1 (Sirtuin 1)/AMPK/PGC1*α* (peroxisome proliferator-activated receptor-*γ* coactlvator-1*α*) pathway [59].

In addition to lncRNA-related axes, increasing evidence has suggested that circRNA-miRNA-mRNA axes regulate the apoptosis of cardiomyocytes in IRI of heart disease (see Table 2 for a summary of the studies of circRNAs-miRNAs-mRNAs in IRI). A letter published in 2019 speculated that the circDLGAP4/miR-143 pathway may be a potential regulator of cardiomyocyte apoptosis in myocardial IRI [60]. Intriguingly, a study by Chen et al. confirmed this speculation [61]. The authors found that the overexpression of circDLGAP4 effectively restored the decreased expression of the circRNA HECT domain E3 ubiquitin protein ligase 1 (HECTD1) resulting from miR-143 inhibition in human umbilical vein endothelial cells, which contributed to the attenuation of endothelial cell dysfunction induced by IRI by increasing cell viability and decreasing cell apoptosis and migration [61]. The circRNA sodium/calcium exchanger 1 (circNCX1) was upregulated in both H9C2 cells and neonatal rat myocardial cells after treatment of H_2_O_2_ or H/R and promoted the production of ROS and myocardial cell apoptosis induced by IRI by targeting miR-133a-3p and leading to overexpression of proapoptotic gene cell death-inducing protein (CDIP1) in a myocardial I/R mouse model [62]. In addition, mitochondrial fission and apoptosis-related circRNA (MFACR) regulated mitochondrial fission and apoptosis in the heart by directly targeting and downregulating miR-652-3p, which blocked mitochondrial fission and reduced cardiomyocyte cell death by suppressing MTP18 translation. Consequently, the knockdown of MFACR attenuated the I/R-induced upregulation of mitochondrial fission, apoptosis, and MI size [63]. Furthermore, the circRNA serine/threonine-protein kinase tousled-like 1 (circTLK1) was prominently upregulated in a myocardial IRI mouse model, leading to significantly increased cardiomyocyte apoptosis by its activity as a sponge of miR-214. miR-214 abolished the negative effects of receptor-interacting serine/threonine-protein kinase 1 (RIPK1) in myocardial IRI, including an impaired cardiac function index, distensible infarct area, and cell apoptosis. These results indicate that the circTLK1/miR-214/RIPK1 axis plays a crucial role in myocardial IRI, which may provide therapeutic targets for treatment [64]. Chang et al. [65] reported that circ_100338 regulates angiogenesis and metastasis of myocardial I/R through miRNA-200a-3p/FUS.

As described above, many lncRNA/circRNA-miRNA-mRNA axes mediate cell apoptosis of cardiomyocytes in IRI. Apoptosis is induced by the “extrinsic” and “intrinsic” pathways, and there are multiple biochemical and functional linkages between the two pathways. I/R, as cytotoxic stimuli, induces the translocation and integration of prodeath members of the Bcl2 protein family (e.g., Bax and Bak) into the outer mitochondrial membrane [18]. However, ischemia per se is not sufficient for activation of Bcl2 proteins because many are redox sensitive, requiring the oxidative stress that is evoked by reperfusion. I/R-induced cell death is reduced in animals treated with pan-caspase inhibitors, providing additional support for the notion that apoptosis contributes to the death of cardiac myocytes [66, 67]. While such observations might lead to the proposal that targeting caspases may be an important therapeutic means to reduce I/R injury, caspase inhibition may not be ideal because other aspects of mitochondrial function will be adversely affected. Therefore, these lncRNA/circRNA-miRNA-mRNA axes may be a suitable alternative in the regulation of cardiomyocyte apoptosis and represent potential therapeutic targets of cell apoptosis during cardiac I/R injury.

#### 2.1.2. lncRNA-miRNA-mRNA Axis Regulates Autophagy of Cardiomyocytes in IRI

Autophagy is a highly conserved catabolic process that provides organelle quality control and generates intracellular nutrients from lysosomal processing of cellular structures [68]. Yu et al. first speculated that MALAT1 may negatively regulate the expression of miR-204, which could increase the autophagy of cardiomyocytes and myocardial IRI [69]. Furthermore, MALAT1 promoted OGD/R-induced H9C2 cell injury by sponging miR-20b to enhance Beclin1-mediated autophagy [70]. miR-204 was found to regulate autophagy through LC3-II during myocardial I/R [71]. Based on the above conclusions, Wang et al. [72] ultimately speculated that the MALAT1/miR-204/LC3-II pathway may be an important regulatory axis of autophagy in myocardial IRI. Nonetheless, further experimental evidence is needed to confirm this possibility. The lncRNA AK139128 is involved in the regulation of autophagy and apoptosis in myocardial IRI by targeting the miR-499/FOXO4 axis [73]. In addition, silencing AK139328 by siRNA significantly enhanced miR-204-3p expression and suppressed cardiomyocyte autophagy, thereby attenuating myocardial IRI in diabetic mice [74]. The lncRNA AK088388 competitively binds to miR-30a, which promotes the expression of autophagy-related proteins, Beclin1 and LC3-II, and eventually leads to cell damage in myocardial IRI [75]. The lncRNA autophagy-promoting factor (APF) mediates the conduction of autophagic-related signals in cardiomyocytes and competitively binds to miR-188-3p, thus indirectly upregulating the expression of ATG7 (Autophagy-related gene 7) and affecting autophagic cell death and MI [76]. Furthermore, Chen et al. [77] found that I/R induced a significant increase in miR-128 associated with a decrease in UCA1 and HSP70, which was reversed by morphine postconditioning treatment that also ameliorated infarct size and cell autophagy. This result suggested that morphine postconditioning treatment preserved myocardium from injury by mediating the UCA1/miR-128/HSP70 pathway.

Autophagy is actually a cell survival mechanism rather than a cell death process and can be activated by I/R-related conditions (e.g., energy deprivation, oxidative stress, and ER stress) [78]. However, uncontrolled autophagy ultimately leads to cell death and may contribute to I/R injury. Autophagy is involved in myocardial IRI through a dual regulation: protection of myocardial cell death during the myocardial ischemia stage and prevention of myocardial cell death during the myocardial reperfusion stage. Inhibition of autophagy has been shown to amplify I/R-induced damage [78, 79], while pharmacologic stimulation of autophagy confers protection against I/R [80, 81].

#### 2.1.3. lncRNA-miRNA-mRNA Axis Regulates Necrosis of Cardiomyocytes in IRI

Distinct from the programmed property of apoptosis and autophagy, necrosis is an uncontrolled process that occurs randomly under the condition of overwhelming stress and contributes to the “accidental” death of the cell [2]. The lncRNA necrosis-related factor (NRF) functions by directly binding to miR-873 and regulates RIPK1/RIPK3 expression and necrosis. Necrosis in cardiomyocytes and MI induced by IRI is attenuated when the expression of NRF is knocked down. Furthermore, p53 regulates cell necrosis in the heart by targeting NRF, miR-873, and the RIPK1/RIPK3 axis in the necrotic cascades [82]. The death program of cytokine-induced necrosis in myocardial IRI was further aggravated when H19 was downregulated by RNA interference. Further research found that H19 decreased the necrotic cell death of cardiomyocytes by interfering with the expression of miR-103/107 that promoted cell necrosis in a cellular model treated with H_2_O_2_ and in a myocardial IR mouse model by inhibiting the expression of FADD (Fas-associated protein with death domain) [83]. Further studies should explore how the H19-miR-103/107-FADD pathway is involved in the intricate necrotic cascade.

Necrosis is one of the main forms of cell death that is most prominent in the I/R heart. Cells can be driven to necrosis by I/R via the activation of at least three separate signaling pathways: necroptosis, mitochondrial permeability transition-dependent regulated necrosis, and parthanatos [84, 85]. Although these lncRNA-miRNA-mRNA axes mediate necrosis of cardiomyocytes in IRI, how these axes integrate into the complex necrotic cascade and the relationships with other necrotic-related factors remain to be studied.

#### 2.1.4. lncRNA-miRNA-mRNA Axis Regulates Inflammation of Cardiomyocytes in IRI

The I/R-induced inflammatory response in most organs has been termed sterile inflammation because of the absence of microorganisms. However, similar to the response to all kinds of microorganism pathogens, sterile inflammation derived from IRI is characterized by the recruitment of peripheral immune cells to the injured tissue sites, accompanied with the production and release of cytokines and chemokines [86]. As discussed above, MALAT1 plays vital roles in I/R pathogenesis by mediating cell death. Some studies speculated that MALAT1 regulates the inflammatory response in myocardial IRI via targeting different targets. One of the first studies showed that MALAT1 upregulates NLRP3 inflammasome expression potentially by sponging miR-133 in the I/R-injured heart [87]. Moreover, Ruan et al. [88] speculated that MALAT1 may aggravate the inflammation response through regulating PTGS2 (Prostaglandin-endoperoxide synthase 2) by targeting miR-26b in myocardial IRI. MALAT1/miR-203 was also considered important in I/R by increasing cardiomyocyte inflammation and myocardial injury [89].

Inflammation plays a prominent role in the reperfusion component of total tissue injury in I/R, which is characterized by leukocyte trafficking to ischemic sites that occur primarily during reperfusion, and I/R-induced leukocyte infiltration contributes to a large number of pathologic processes [86]. Furthermore, leukocyte endothelial cell adhesive interactions, which precipitate the microvascular complications and tissue injury induced by reperfusion, are one of the earliest signs of tissue dysfunction and injury elicited by I/R [90, 91]. Multiple factors are involved in the dynamic regulation of inflammation in myocardial IRI upon AMI, and thus inhibition of the inflammatory response may be a potential therapeutic strategy [92]. Therefore, it may be possible to reduce or prevent the production of IRI by interfering with the inflammatory response produced by these axes.

### 2.2. Brain

Acute ischemic stroke (AIS) is a pathological process that starts with local cerebral vascular occlusion and is accompanied by a series of changes in cellular behaviors, leading to sudden local brain dysfunction [93]. The effective treatment for AIS is to restore blood flow, which can lead to reperfusion injury. Cerebral IRI often occurs in stroke and cardiac arrest and induces neuronal damage. Increasing evidence demonstrates that ischemia is often associated with a series of neurological disorders, such as hypoxia, oxidative stress, and inflammatory responses, which eventually lead to acute necrosis, apoptosis, and autophagy of ischemic brain cells [94]. In recent years, ncRNAs were found to play important roles in physiopathological processes related to stroke (see Table 3 for a summary of the studies on lncRNAs-miRNAs-mRNAs in cerebral IRI).

#### 2.2.1. lncRNA-miRNA-mRNA Axis Regulates Apoptosis of Nerve Cells in IRI

In cerebral IRI, the main mechanism of brain injury mainly involves apoptosis of nerve cells. MALAT1 and TUG1 may exhibit similar roles in cerebral IRI. MALAT1 and TUG1 were significantly upregulated in both a middle cerebral artery occlusion/reperfusion (MCAO/R) model and an OGD/R model, and knockdown of both lncRNAs increased cell viability and reduced apoptosis. Furthermore, MALAT1 and TUG1 promote cerebral IRI by targeting miR-145/AQP4, which may be a potential treatment strategy for ischemic stroke [95, 96]. A previous report showed that the H19/miR-138-5p/p65 axis critically regulates inflammation and neurological function in cerebral IRI. H19 inhibited cell proliferation, increased cell apoptosis, and aggravated inflammation after OGD/R stimulation in vitro. And H19 also deteriorated inflammation and neurological function in a transient middle cerebral artery occlusion (tMCAO) rat model in vivo [97]. The H19/miR-19a/Id2 axis regulates hypoxia-induced neuronal apoptosis, and inhibition of this axis may serve as a novel therapeutic strategy for ischemic brain injury [98]. Previous reports have established a role and mechanism of metformin in shock-related brain injury [99]. Zeng et al. [99] found that H19 modified oxidative stress under OGD/R by binding to miR-148a-3p to upregulate Rock2 (Rho-associated protein kinase 2) expression, which was reversed by metformin. The study ultimately showed that metformin plays a neuroprotective role by regulating ischemic stroke-induced oxidative stress injury through H19/miR-148a-3p/Rock2. In addition, upregulation of Rian reduced the OGD-induced N2a cell apoptosis, reduced the miR-144-3p/GATA3-mediated infarct size, and ameliorated the neurological score [100]. Other signal axes mediate brain damage by affecting neuronal apoptosis, including the Rian/miR-144-3p/GATA3, Gm11974/miR-766-3p/NR3C2, Oprm1/miR-155/GATA3, AK038897/miR-26-5p/DAPK1, CHRF/miR-126/SOX6, SNHG16/miR-106b-5p/LIMK1, and KCNQ1OT1/miR-9/MMP-8 axes [100–106].

#### 2.2.2. lncRNA-miRNA-mRNA Axis Regulates Autophagy of Nerve Cells in IRI

By regulating the miR-26b/ULK2 and miR-30a/Beclin1 pathways, MALAT1 promoted OGD/R-induced nerve cell autophagy and ischemic injury [107, 108]. Yu et al. [109] showed that the lncRNA KCNQ1OT1 promotes I/R-induced autophagy and reduces cell viability by regulating the miR-200a/FOXO3/ATG7 axis. Furthermore, KCNQ1OT1 was significantly increased in the plasma of patients with AIS, and its expression was positively correlated with the severity of stroke, which implied that KCNQ1OT1 may be a diagnostic biomarker or severity evaluation indicator.

#### 2.2.3. lncRNA-miRNA-mRNA Axis Regulates Inflammation of Nerve Cells in IRI

SNHG12 (Small nucleolar RNA host gene 12)/miR-199a inhibits BMECs and N2a cell death and the inflammatory response and promotes angiogenesis after OGD/R by targeting different mRNAs [110, 111]. Interestingly, SNHG1/miR-199a promoted BMEC survival, migration, and tube formation under OGD/R by elevating the expression of HIF-1*α* and VEGF [112]. In addition, the SNHG14 (Small nucleolar RNA host gene 14)/miR-136-5p/ROCK1 axis contributed to neurological impairment and inflammatory response in cerebral ischemia stroke [113]. Some small nucleolar RNA host genes (SNHG) and miR-199a are important mediators in cerebral IRI. Furthermore, MEG3 promoted pyroptosis and inflammation under OGD/R and facilitated Caspase-1 signaling by binding miR-485 to increase AIM2 expression [114].

#### 2.2.4. circRNA-miRNA-mRNA Axis in Brain IRI

Lin et al. [115] explored the potential function of circRNAs in the etiopathogenesis of cerebral IRI. The authors investigated the expression profiles of circRNAs between HT22 cells with OGD/R and controls using a circRNA microarray. The results showed that 15 circRNAs were markedly altered in the OGD/R model group. The authors selected mmu-circRNA-015947 for further verification by qRT-PCR. Bioinformatics analysis showed that mmu-circRNA-015947 could bind with miRNAs (mmu-miR-188-3p, mmu-miR-329-5p, mmu-miR-3057-3p, mmu-miR-5098, and mmu-miR-683) and thereby elevate the expression of target genes. This research indicates that increasing the level of mmu-circRNA-015947 might contribute to the process of cerebral IRI and provides a potential strategy for clinical treatment. In addition, three circRNAs (cZNF292, TLK1, and circ_008018) were significantly increased in the MCAO/R mouse model and mouse neurons under OGD/R and knockdown of the circRNAs attenuated neuronal injury during cerebral I/R. These effects were related to cell apoptosis mediated by targeting miR-22/Wnt/b-catenin (under OGD/R), miR-335-3p/TIPARP (under OGD/R and cerebral I/R), and miR-99a/Pi3k/Akt/GSK3b (cerebral I/R) [116–118]. Furthermore, the expression levels of circTLK1 in patients with AIS were notably increased in comparison with healthy controls. Interestingly, the expression levels of circTLK1 in plasma from patients with stroke were associated with lesion localization and infarct volumes [117]. Han et al. [119] found that the levels of circHECTD1 were elevated in tMCAO mouse stroke models and in plasma of AIS patients. Knockdown of circHECTD1 contributed to reduction in infarct areas, attenuation of neuronal deficits, and amelioration of astrocyte activation via mediating autophagy in tMCAO mice through miR-142/TIPARP. Thus, circHECTD1 was considered a new biomarker and therapeutic target for stroke. Jiang et al. [120] found that an upregulated circRNA, antisense noncoding RNA in the INK4A locus (circANRIL), inhibited OGD/R-induced HBMEC proliferation and promoted cell apoptosis and phosphorylation of p65 and I*κ*B*α*, which were abrogated by miR-622. These results demonstrated that circANRIL aggravated OGD/R-induced injury in HBMECs by mediating the NF-*κ*B pathway through sponging miR-622.

### 2.3. Liver

#### 2.3.1. lncRNA-miRNA-mRNA Axis Regulates Apoptosis of Hepatocytes in IRI

Liver IRI, which occurs in hemorrhagic shock, resection, and transplantation, starts with local ischemic insult, followed by inflammation-mediated reperfusion injury [121] (see Table 4 for a summary of the studies of lncRNAs-miRNAs-mRNAs in other diseases with IRI). A recent study investigated the role of the lncRNA Gm4419 in hepatic I/R [122]. The authors found that Gm4419 was upregulated in hepatic IRI rats, and knockdown of Gm4419 aggravated I/R-induced liver damage in hepatic IRI rats. Gm4419 promoted H/R-induced apoptosis by sponging miR-455 and regulating SOX6 in BRL-3A cells. Therefore, Gm4419 accelerated hepatic IRI by interacting with the miR-455/SOX6 axis. Huang et al. [123] reported that the expression levels of MEG3 and Nrf2 were decreased in hepatic I/R mice and in HL7702 cells with H/R treatment, while miR-34a was increased. Overexpression of MEG3 inhibited apoptosis and affected the production of ROS *in vitro*, which was abrogated by miR-34a inhibitor treatment. MEG3 overexpression ameliorated the hepatic function of hepatic I/R mice and significantly reduced the level of serum ALT and AST. These results indicated that MEG3 protected hepatocytes from hepatic IRI through miR-34a/Nrf2. Furthermore, Dai et al. [124] found that the levels of AK054386, miR-199, and CHOP (C/EBP homologous protein) were elevated, decreased, and elevated in hepatic IRI models, respectively. Overexpression of AK054386 promoted cell apoptosis in the BNL-CL2 IRI cell model and CHOP expression, which were rescued by miR-199 overexpression. These results suggest that AK054386 plays a crucial role in hepatic IRI via miR-199 by mediating the ERS pathway.

#### 2.3.2. lncRNA-miRNA-mRNA Axis Regulates Autophagy of Hepatocytes in IRI

Autophagy has been proven to be involved in hepatic IRI [125, 126]. Liver IRI induces elevated levels of HOTAIR and ATG7 and increases autophagy, which is attenuated by the knockdown of HOTAIR. In addition, HOTAIR acts as a ceRNA for miR-20b-5p and increases the expression of ATG7. These results indicated that the HOTAIR/miR-20b-5p/ATG7 axis plays a crucial role in hepatic IRI via autophagy [127].

#### 2.3.3. circRNA-miRNA-mRNA Axis in Liver IRI

Zhang et al. [128] first examined circRNA expression profiles during hepatic IRI by microarray hybridization analysis and found that circRNAs are closely associated with hepatic IRI and ischemic postconditioning (IPO). The analyses revealed that the expression of 1599 circRNAs was altered, including 213 upregulated and 493 downregulated circRNAs, between the I/R group and the control group. In a comparison of the IPO group with the I/R group, the results revealed that 641 circRNAs were upregulated and 252 circRNAs were downregulated. Moreover, the ceRNA network, including 6 circRNAs, 47 miRNAs, and 90 mRNAs, illustrated that the “housekeeping” function of circRNAs is abnormally regulated in hepatic IRI. The mmu_circRNA_005186/miR-124-3p/Epha2 axis was chosen for further study after qRT-PCR validation. Silencing of mmu_circRNA_005186 moderated lipopolysaccharide-induced inflammation by elevating miR-124-3p and reducing Epha2, which suggests that the mmu_circRNA_005186/miR-124-3p/Epha2 axis might play an important role in hepatic IRI.

### 2.4. Kidney

#### 2.4.1. lncRNA-miRNA-mRNA Axis in Renal IRI

Renal IRI, which contributes greatly to AKI, is one of the most critical issues for many clinical situations, including renal transplantation, nephrectomy, sepsis, and repair of suprarenal aneurism [129–131]. Hu et al. [132] used a microarray assay to find that 2218 genes are differentially expressed in renal IRI, including 1103 upregulated genes and 1115 downregulated genes. The authors further reported that both the dysregulated lncRNA MALAT1 and miR-139-5p participated in IRI and were closely associated with cell proliferation [133]. In addition, Geng et al. [134] revealed that GAS5, which is prominently elevated in renal IRI, was inhibited by delayed ischemic preconditioning (IPC), and GAS5 levels were increased by knocking down miR-21 before IPC *in vivo*. A negative regulatory relationship was detected between miR-21 and TSP-1 (Thrombospondin 1) both *in vitro* and *in vivo*. The role of GAS5 in H/R-induced cell apoptosis was already illuminated in the authors' previous work [135]. Together, these results indicated that GAS5 promoted apoptosis by functioning as a miR-21 sponge and downregulating TSP-1 in renal IRI.

NEAT1 was reported to facilitate renal tubular epithelial apoptosis induced by H/R via binding to miR-27a-3p, and reciprocal inhibition was detected between NEAT1 and miR-27a-3p. Furthermore, miR-27a-3p was expressed at low levels while NEAT1 was overexpressed in AKI patients compared with healthy controls [136]. Xu et al. [137] confirmed that TUG1 silencing attenuates inflammation and apoptosis via binding to miR-449b-5p and downregulating HMGB1 and MMP-2 expression in renal IRI. In addition, Tang et al. [138] reported that loss of XIST and PDCD4 enhanced CoCl2-processed HK-2 cell proliferation and repressed cellular apoptosis, which was reversed by miR-142-5p. Furthermore, knockdown of LINC00520 protected against AKI both *in vitro* and *in vivo* by mediating PI3K/AKT through miR-27b-3p/OSMR [139].

#### 2.4.2. circRNA-miRNA-mRNA Axis in Renal IRI

Huang et al. [140] reported that the circRNA yes-associated protein 1 (circYAP1) expression was downregulated in AKI patients as well as in I/R-treated HK-2 cells. circYAP1 overexpression promoted cell growth and attenuated the secretion of inflammatory factors and ROS generation in HK-2 cells under I/R treatment. In addition, the authors found that circYAP1 was a functional sponge for miR-21-5p that reversed the inhibitory effects of circYAP1 on cell injury. Furthermore, circYAP1 inhibits miR-21-5p to activate the PI3K/AKT/mTOR pathway. These results revealed that circYAP1 activates the PI3K/AKT/mTOR signal pathway and protects HK-2 cells against renal IRI via binding to miR-21-5p.

### 2.5. Other IRI

#### 2.5.1. lncRNA-miRNA-mRNA Axis in Retinal IRI

Retinal IRI, a cause of irreversible visual damage, occurs with glaucoma, diabetic retinopathy, and retinal vascular occlusive disorders [141]. One study [142] reported that the Mbd2-AL1 (Methyl-CpG-binding domain protein 2-associated long noncoding RNA 1)/miR-188-3p/Traf3 axis plays an important role in mediating retinal ganglion cell apoptosis and visual function in Mbd2-KO mice. The results implied that the lncRNA Mbd2 may be a novel therapeutic target for retinal ischemic diseases. In addition, there may be many similarities between the roles of miR-21/PDCD4 in retinal IRI and cerebral IRI. Wan et al. [143] reported that I/R-mediated H19 overexpression facilitates NLRP3/6 inflammasome imbalance and leads to retinal microglial pyroptosis, excess cytokine secretion, and neuronal death. MEG3 aggravates ischemic damage and deteriorates overall neurological functions by binding to miR-21 and downregulating PDCD4, which mediates sterile inflammation and neuronal lesions in cerebral IRI [144].

#### 2.5.2. lncRNA-miRNA-mRNA Axis in Testicular IRI

Li et al. [145] examined the potential role of MALAT1 in testicular IRI. The authors demonstrated that the expression level of MALAT1 in animal testis samples and GC-1 cells was elevated. Overexpression of MALAT1 promoted apoptosis and inhibited proliferation as testicular IRI progressed. Furthermore, MALAT1 inhibited expression of miR-214 and positively regulated TRPV4 (Transient receptor potential vanilloid 4) expression. These results indicated that the MALAT1/miR-214/TRPV4 axis plays an important role in testicular IRI by mediating cell apoptosis and proliferation.

#### 2.5.3. lncRNA-miRNA-mRNA Axis in Spinal Cord IRI

Liu et al. [146] found that inhibition of the lncRNA cancer susceptibility candidate 7 (CasC7) promoted cell apoptosis in SH5Y-SY cells under OGD/R treatment and increased infarct size in spinal cord IRI rats through miR-30c/Beclin1, which was reversed by NaSH preprocessing. The study concluded that hydrogen sulfide saves the spinal cord from IRI by the CasC7/miR-30c/Beclin1 axis. The expression of MALAT1 and Bcl2 was suppressed while miR-204 was upregulated in a rat spinal cord IRI model and hypoxia-induced neurocyte lines [147]. Furthermore, knockdown of MALAT1 promoted cell apoptosis, which was associated with downregulation of Bcl2 and upregulation of miR-204. MALAT1-treated spinal cord IRI rats also showed lower motor deficit index scores. Therefore, these results indicated that MALAT1 plays a neuroprotective role in spinal cord IRI rats by binding miR-204/Bcl2.

#### 2.5.4. lncRNA/circRNA-miRNA-mRNA Axis in Intestinal IRI

Zou et al. [148] revealed that H19 overexpression increased the level of miR-675, which in turn inhibited the expression of ZO-1 (Zonula occludens 1) and E-cadherin, leading to dysfunction of the epithelial barrier. These effects were reversed by upregulation of the RNA-binding protein HuR in H19-overexpressing cells. These results revealed that H19 and HuR act upon each other, and H19 mediates the intestinal epithelial barrier function via the miR-675/ZO-1/E-cadherin axis. Feng et al. [149] explored the expression profiles of circRNAs after intestinal I/R with or without IPO and investigated the underlying mechanisms of IPO associated with the altered circRNAs. The authors identified 62 circRNAs and 521 mRNAs differentially expressed in the intestinal I/R group compared with the sham group, as well as 33 circRNAs and 303 mRNAs that were altered between the IPO group and I/R group. Two circRNAs, circRNA_012412 and circRNA_016863, were identified as closely related to the protective mechanisms of IPO. Ultimately, four pathways, circRNA_012412/miR-7649-3p/Sertad1 (SERTA domain-containing protein 1), circRNA_012412/miR-3473c/Sertad1, circRNA_012412/miR-6991-3p/Nudcd1 (NudC domain-containing protein 1), and circRNA_012412/miR-6991-3p/Jam2 (Junctional adhesion molecule B), were constructed based on the TargetScan, miRanda, and miRDB databases. These circRNA regulatory pathways may be closely associated with endogenous protective signaling in IPO during intestinal I/R and warrant further investigation. The study was the first to fully describe the circRNA expression profiles during intestinal I/R, and the results showed that IPO was associated with altered circRNAs, which provides a new perspective to clarify how IPO protects against intestinal IRI.

## 3. Conclusions and Future Perspectives

Over the past few decades, ncRNAs have been found to play complex roles in the development and gene regulatory processes of many diseases. However, understanding of the mechanisms of lncRNAs and circRNAs in different organs and dysfunctional states and their potential as therapeutic targets or diagnostic markers of IRI is still in its infancy. Furthermore, the interactions of the lncRNAs/circRNAs-miRNAs-mRNAs are complex and dynamic. First, a specific lncRNA or circRNA plays a variety of roles in IRI of different organs by targeting different miRNAs/mRNAs. The expression levels of lncRNAs and circRNAs in animal and cell models seem to be similar as in patient serum and were associated with the severity of the disease, providing evidence for clinical diagnosis and prognosis. Second, the same lncRNA or circRNA can play a contradictory role in IRI of the same organ, potentially contributing to other molecules in response to stress conditions. For example, H19 showed opposite expression levels and effects in myocardial IRI, and future studies should investigate the various regulatory mechanisms. Third, some lncRNAs and circRNAs can target the same miRNAs/mRNAs in different organs. For instance, MEG3 and H19 both performed their functions in cerebral IRI and in retinal IRI, respectively, by sponging miR-21 and targeting PDCD4 to mediate cell apoptosis [143, 144]. Finally, some drugs and treatments play an important role in IRI through the functions of these axes. For example, metformin protects against oxidative stress injury in cerebral IRI by affecting the H19/miR-148a-3p/Rock2 axis [99], while the cardioprotective effects of fentanyl in cardiac IR appeared to be abrogated by the MALAT1/miR-145/Bnip3 axis [22]. Furthermore, IPO can attenuate liver IRI and exhibit significant intestinal protection through circRNAs-miRNAs-mRNAs [128, 149], and the renal protection of delayed IPC involves preconditioning-induced upregulation of miR-21 and downregulated expression of GAS5 and TSP-1 [134]. Although these studies have shown some benefit, they have not been able to identify a specific effective protocol of IPC/IPO for a clinical study, perhaps because it has been very difficult to systematically illuminate the underlying complex mechanisms of ncRNAs. Therefore, further investigations focusing on revealing the specific molecular mechanisms of ncRNAs in the development of IRI and abnormal conditions are needed.

Although cardiac magnetic resonance imaging remains the gold standard to assess the consequences of acute IRI in AMI patients in terms of MI size and adverse left ventricular remodeling, this technique is limited in terms of ease of access and availability of skilled personnel [150]. However, the presence of ncRNAs in the serum plasma has suggested that these molecules may serve as biomarkers in AMI patients or patients with myocardial IRI [34, 41, 117, 119, 151]. Although plasma contains RNases, circulating ncRNAs, especially lncRNAs, have been shown to be stable in this environment, which indicates that they are relatively resistant to nucleolytic degradation and making them potentially useful as circulating biomarkers for AMI. Furthermore, ncRNA or the antisense molecules may be delivered to the ischemic heart using several approaches including intravenous or intramyocardial injections or carriers such as viruses, nanoparticles, or exosomes. Zhang et al. [152] found that hypoxia modified the expression of several miRNAs in exosomes secreted by H9c2 cells, and these exosomal miRNAs protected H9c2 cells against simulated IRI and prevented apoptosis through HIF-1, TNF, MAPK, and mTOR signaling pathways. Some miRNAs or lncRNAs that are downregulated following AMI are known to be beneficial for cardioprotection, and one therapeutic strategy is to deliver ncRNA mimics targeting these miRNAs or lncRNAs to the ischemic heart. Furthermore, an alternative approach to upregulate cardioprotective miRNAs may involve a small-molecule therapeutic strategy. Therefore, further research on these ncRNAs involved in IRI of different organs is urgently needed, and these studies may lead to the identification of other unknown signaling pathways or reactions in IRI. This research will also help elucidate the contribution of ncRNAs to the pathophysiology of IRI and subsequent clinical outcomes and provide support for them as potential markers or therapeutic targets for IRI.

In summary, the lncRNA/circRNA-miRNA-mRNA axes have multiple roles in IRI, and factors in these axes may function as diagnostic markers and therapeutic targets. Several studies involving the lncRNA/circRNA-miRNA-mRNA axis are imperative and continuous efforts will be necessary for us to integrate these axes with clinical practice.

## Figures and Tables

**Table 1:** The lncRNA-miRNA-mRNA axis in myocardial I/R.

Models	Species	Cell dysfunction	Expression	lncRNA	miRNA	mRNA	Function	Mechanism	Relationship	Prediction tool	Ref
HL-1 cells and MCM under H/R treatment	Mouse	Autophagy	↑	AK088388	miR-30a	Beclin1/LC3	Interfering AK088388 can promote the viability of H/R cardiomyocytes, reduce lactate dehydrogenase release, and reduce apoptosis	miR-30a had binding sites on AK088388	NR	n/a	[75]
I/R in rats and newborn rats' primary cardiomyocytes under H/R treatment	Rat	Autophagy; apoptosis	↑	AK139128	miR-499	FOXO4	Knockdown of AK139128 impressively alleviates cardiomyocyte autophagy and apoptosis	There are several complementary binding sites within miR-499 and AK139128	NR	starBase v3.0; TargetScan	[73]
IRI in mice and mice primary cardiomyocytes under H/R treatment	Mouse	Autophagy; apoptosis	↑	AK139328	miR-204-3p	CK; CK-MB; LDH	Knockdown of lncRNA AK139328 relieved myocardial I/R injury in DM and inhibited cardiomyocyte autophagy as well as apoptosis of DM	Modulated miR-204-3p directly	NR	n/a	[74]
IRI in mice and mice primary cardiomyocytes under A/R	Mouse	Autophagy	↑	APF	miR-188-3p	ATG7	APF conveys the autophagic signal in cardiomyocytes. APF participates in mediating the signal for autophagy and cell death in the heart	Is able to directly bind to miR-188-3p and regulate its activity	NR	n/a	[76]
I/R in mice and mice primary cardiomyocytes under anoxia	Mouse	Apoptosis	↓	CARL	miR-539	PHB2	CARL is able to prevent mitochondrial fission, apoptosis, and myocardial injury in myocardial infarction	Can act as an endogenous miR-539 sponge	NR	n/a	[43]
H9C2 cells under H/R treatment	Human; rat	Proliferation; apoptosis	↓	FTX	miR-410-3p	Fmr1	Overexpression of FTX relieved the damage caused by H/R treatment in H9c2 cells	A sponge for miR-410-3p	NR	LncBase predicted v.2	[41]
I/R in mice and mouse primary cardiomyocytes under H_2_O_2_ treatment	Mouse	Apoptosis	↓	FTX	miR-29b-1-5p	Bcl2l2	Enhanced expression of FTX inhibits cardiomyocyte apoptosis	Functions as endogenous sponge for miR-29b-1-5p	NR	RNA hybrid	[40]
IRI in rats and H9C2 cells under H/R treatment	Rat	Apoptosis	↑	GAS5	miR-532-5p	PI3K/AKT	Silencing of lncRNA GAS5 was able to attenuate myocardial damage, as cell viability increased and the apoptosis rate decreased	Functioned as a molecular sponge of miR-532-5p	NR	RNA hybrid	[44]
IRI	n/a	n/a	n/a	GAS5	miR-137	Serpina3	lncRNA GAS5 may exacerbate myocardial I/R injury through regulating serpina3 via targeting miR-137	Serve as a ceRNA for miR-137	n/a	n/a	[45]
Neonatal rats' primary cardiomyocytes under H/R treatment	Rat	Apoptosis	↓	H19	miR-29b-3p	cIAP1	H19 mediated the antiapoptotic effect of H/post against H/R-induced injury to aged cardiomyocytes	Participated in the regulation of miR-29b-3p	NR	Bioinformatics analysis	[24]
IR	n/a	Apoptosis	n/a	H19	miR-22-3p	n/a	lncRNA H19/miR-22-3p axis might be a potential regulated signaling pathway of apoptosis in MIRI	Acts as a ceRNA to suppress the activity of miR-22-3p	PR	n/a	[25]
I/R in mice and H9C2 cells under H_2_O_2_ treatment	Mouse	Necrosis	↓	H19	miR-103/107	FADD	H19 mediates necrotic cell death in cardiomyocytes	Is able to directly bind to miR-103/107	NR	n/a	[83]
I/R in mice and NMVCs under H_2_O_2_ treatment	Mouse	Apoptosis	↓	H19	miR-877-3p	Bcl2	Overexpression of H19 alleviated myocardial I/RI of mice and cardiomyocyte injury induced by H_2_O_2_	Functions as a miR-877-3p ceRNA	NR	RegRNA2.0；starBase；TargetScan	[26]
Mouse primary cardiomyocytes under OGD/R condition	Mouse	Viability; apoptosis; inflammation; oxidative stress	↑	H19	miR-675	PPAR*α*	Knockdown of H19 significantly reduced infarct size, increased left ventricular systolic pressure, and decreased left ventricular end-diastolic pressure in a mouse model of myocardial I/R	Is a precursor of miR-675	PR	n/a	[27]
I/R in mice	Mouse	Fibrosis; apoptosis	↑	HIF1A-AS1	miR-204	SOCS2	Downregulation of HIF1A-AS1 alleviates ventricular remodeling and improve cardiac function in mice after myocardial I/R injury	Adsorbs miR-204 as a ceRNA	NR	RNA22	[46]
IRI in mice and H9C2 cells under H/R treatment	Mouse	Apoptosis	↑	HOTAIR	miR-451	Cab39	Hotair overexpression prevented I/R-induced oxidative stress, cardiac myocyte apoptosis, and cardiac dysfunction	Contributed to Hotair-mediated miR-451 inhibition	NR	n/a	[38]
IR	n/a	n/a	↓	HOTAIR	miR-126	n/a	Circulating HOTAIR/miR-126 axis maybe a potential biomarker and risk factor predictor for MI/R injury	Act as a ceRNA	n/a	n/a	[39]
H9C2 cells under H_2_O_2_ treatment	Rat	Apoptosis; proliferation	↓	HOTAIR	miR-125	MMP-2	Repression of HOTAIR accelerates H9c2 cells injury in response to oxidative stress	miR-125 is a target of HOTAIR	NR	Bioinformatic analysis	[37]
IRI in rats and H9C2 cells under H/R treatment	Rats	Inflammation; apoptosis	↓	HULC	miR-377-5p	NLRP3/Caspase-1/IL-1*β*	HULC modulated myocardial I/R injury in rat models and cardiomyocyte apoptosis in H/R cell models via targeting miR-377-5p through NLRP3/Caspase-1/IL-1*β* pathway	Acted as a ceRNA by sponging miR-377-5p	NR	Bioinformatic analysis	[57]
IRI in mice and mice primary cardiomyocytes under H/R treatment	Mouse	Apoptosis	↑	KCNQ1OT1	miR-204-5p	LGALS3	The downregulation of LGALS3 resulted in the alleviation of myocardial IR injury in mouse models	Bind to miR-204-5p	NR	LncBase v.2; miRDB; DIANA TOOLS	[47]
AMI in rats and HL-1 cells under H/R treatment	Rat	Apoptosis	n/a	MALAT1	miR-125b-5p	NLRC5	Downregulation of MALAT1 attenuated heart damage in an AMI model rat	MALAT1 negatively regulates miR-125b-5p expression	NR	TargetScan	[21]
MI in mice and AC16 cells under hypoxia condition	Mouse	Apoptosis; proliferation	↑	MALAT1	miR-200a-3p	PDCD4	Knockdown of MALAT1 enhanced cell viability, promoted cell cycle progress, and suppressed cell apoptosis	Acted as a ceRNA to sponge miR-200a-3p	NR	starBase v.2.0	[20]
IRI	n/a	Inflammation	↑	MALAT1	miR-26b	PTGS2	Aggravate inflammation response through regulating PTGS2 by targeting miR-26b in MI/R injury	Can act as ceRNA by binding to consensus MREs of miR-26b	n/a	n/a	[88]
IR	n/a	Autophagy	↑	MALAT1	miR-204	LC3-II	MALAT1/miR-204/LC3-II axis is a potential regulated axis of autophagy in myocardial I/R injury	Can sponge miR-204	n/a	n/a	[72]
IR	n/a	Inflammation	↑	MALAT1	miR-203	n/a	lncRNA MALAT1 may increase cardiomyocyte inflammation and myocardial injury during I/R	n/a	NR	n/a	[89]
IR	n/a	Inflammation	↑	MALAT1	miR-133	NLRP3	lncRNA MALAT1 may sponge miR-133 to promote NLRP3 inflammasome expression in ischemia-reperfusion-injured heart	Acted as a ceRNA to inhibit miR-133 action	NR	n/a	[87]
I/R in mice and HL-1 under H/R treatment	Mouse	Apoptosis	↑	MALAT1	miR-145	Bnip3	MALAT1 overexpression reverses cardioprotective effects of fentanyl as indicated by an increase in LDH release and cell apoptosis	Being regulated by miR-145 of MALAT1	NR	n/a	[22]
H9C2 cells under OGD/R condition	Mouse	Autophagy	↑	MALAT1	miR-20b-5p	Beclin1	MALAT1 antagonized the inhibitory effects of miR-20b-5p on Beclin1-related cardiomyocyte autophagy in OGD/R injury	Functions as a ceRNA for miR-20b-5p	NR	n/a	[70]
IR	n/a	Autophagy	↑	MALAT1	miR-204	n/a	lncRNA MALAT1 may increase cardiomyocyte autophagy and myocardial injury during I/R by negatively regulating miR-204 expression	Might serve as a sponge to suppress miR-204 action	NR	n/a	[69]
I/R in mice and mice primary cardiomyocytes under A/R	Mouse	Apoptosis	↓	MDRL	miR-361	miR-484	Knockdown of MDRL induced mitochondrial fission and apoptosis	Is a functional sponge for miR-361	NR	n/a	[42]
I/R in rats and H9C2 cells under H/R treatment	Rat	Apoptosis; proliferation	↑	MEG3	miR-7-5p	PARP1; Caspase-3	Overexpression of MEG3 increased the I/R-induced CK and LDH activities and cell apoptosis and decreased cell proliferation	By directly binding to miR-5-7p	NR	n/a	[48]
I/RI	n/a	n/a	↑	MEG3	miR-223	n/a	Circulating MEG3/miR-223 axis maybe a potential biomarker and risk factor predictor for MI/R injury	Acted as an endogenous sponge for miR-223	n/a	n/a	[49]
MI/R in mice and H9C2 cells under H_2_O_2_ treatment	Mouse	Proliferation	↑	NEAT1	miR-495-3p	MAPK6	Loss of NEAT1 in H9C2 cells could repress the viability and proliferation of cells	Sponges miR-495-3p	NR	n/a	[31]
I/R in rats and H9C2 cells under H/R treatment	Rat	Apoptosis	↑	NEAT1	miRNA-520a	n/a	Knockdown of NEAT1 serves a protective role against H/R-induced cardiomyocyte apoptosis	miR-520a was indicated to directly target NEAT1	NR	Bioinformatics analysis	[32]
H9C2 cells under OGD/R condition	Rat	Proliferation; apoptosis	↑	NEAT1	miR-193a	n/a	Downregulation of lnc-NEAT1 promoted cell proliferation and inhibited cell apoptosis	miR-193a was targeted by lnc-NEAT1 in I/R injury H9c2 cells	NR	starBase; miRcode	[33]
I/R in mice and rat primary cardiomyocytes under H_2_O_2_ treatment	Mouse	Apoptosis	↓	NEAT1	miR-125a-5p	Bcl2l12	Ectopic overexpression of NEAT1 suppresses cardiomyocyte apoptosis induced by hydrogen peroxide	Functions as miR-125a-5p sponge	NR	starBase v2.0	[36]
IR	n/a	n/a	↑	NEAT1	miR-27b	PINK1	lncRNA NEAT1 may aggravate diabetic MI/R injury	Can sponge miR-27b	n/a	n/a	[35]
IR in mice and newborn rat primary cardiomyocytes under hypoxia condition	Mouse	Proliferation; apoptosis	↑	NEAT1	miR-378a-3p	ATG12	lncRNA NEAT1 significantly promoted cell proliferation and migration of cardiomyocytes	Was capable of targeting miR-378a-3p	NR	RNA hybrid	[34]
I/R in mice and mice primary cardiomyocytes under H_2_O_2_ treatment	Mouse	Necrosis	↑	NRF	miR-873	RIPK1/RIPK3	Knockdown of NRF antagonizes necrosis in cardiomyocytes and reduces necrosis and myocardial infarction upon I/R injury	As an endogenous sponge RNA	NR	n/a	[82]
IRI in rats and H9C2 cells under H/R treatment	Rats	Apoptosis	↓	Oprm1	miR-30b-5p	CSE	Overexpression of lncRNA Oprm1 mitigated MIRI and preserved the cardiac function in vivo	Competitively combines with miR-30b-5p	NR	Bioinformatic analysis	[58]
IRI in rats and H9C2 cells under OGD/R condition	Rats	Apoptosis	↓	OIP5-AS1	miR-29a	SIRT1/AMPK/PGC1*α*	OIP5-AS1 overexpression alleviated reactive oxygen species-driven mitochondrial injury and consequently decreased apoptosis in MI/R rats and H9c2 cells exposed to OGD/R	Acted as a ceRNA of miR-29a	NR	DIANA-LncBase; starBase	[59]
AMI in rats	Rat	Apoptosis	↑	PINT	miR-208a-3p	JUN	Low expression of LINC-PINT could suppress myocardial infarction apoptotic cells	Could sponge miR-208a-3p	NR	n/a	[50]
H9C2 cells under hypoxia condition	Rat	Apoptosis	↑	RMRP	miR-206	ATG3	Upregulation of RMRP may aggravate myocardial I/R injury	Sponging miR-206	NR	n/a	[51]
IRI in rats and HCMs under H/R treatment	Rats	Inflammation; apoptosis	↑	ROR	miR-124-3p	TRAF6	Overexpression of ROR further enhanced the H/R-induced inflammation and cell apoptosis	Sponged and negatively regulated miR-124-3p	NR	Bioinformatics analysis	[56]
MI/R in mice and HUVECs under H/R treatment	Human; mouse	Proliferation	↑	SNHG1	miR-140-3p	HIF-1*α*/VEGF	SNHG1 upregulation under H/R increased HUVEC proliferation, tube formation, and cell migration	Functioned as a ceRNA of miR-140-3p	NR	TargetScan	[55]
IR in mice and neonatal mice primary cardiomyocytes under H_2_O_2_ treatment	Mouse	Cell viability; apoptosis	↑	TUG1	miR-132-3p	HDAC3	Knocking down TUG1 significantly improved viability, inhibited apoptosis, and reduced ROS production in H_2_O_2_-stressed cardiomyocytes in vitro, and alleviated I/R-induced AMI in vivo	Sponged miR-132-3p	NR	TargetScan	[52]
IRI in mice and mice primary cardiomyocytes under H_2_O_2_ treatment	Mouse	Autophagy; apoptosis	↑	TUG1	miR-142-3p	HMGB1/Rac1	Inhibition of TUG1 and overexpression of miR-142-3p inhibited cell apoptosis and autophagy in cardiomyocytes	Sponging miR-142-3p	NR	n/a	[53]
IRI in rats and H9C2 cells under H/R treatment	Rat	Autophagy	↓	UCA1	miR-128	HSP70	UCA1/miR-128 mediated the mechanism of MPostC on autophagy and myocardial injury	Could bind with miR-128	NR	n/a	[77]
IRI	n/a	Apoptosis	↓	UCA1	miR-143	n/a	lncRNA UCA1 interferes with miR-143 expression to modulate cardiomyocyte apoptosis in myocardial I/R injury	lncRNA UCA1 directly interactS with miR-143	NR	n/a	[54]

↑: the upward arrow indicates increased expression of lncRNAs; ↓: the downward arrow indicates decreased expression of lncRNAs; n/a: not applicable; NR: lncRNAs negatively regulate miRNAs; PR: lncRNAs positively regulate miRNAs; RC: reciprocal correlations between lncRNAs and miRNAs; IRI: ischemia-reperfusion injury; AMI: acute myocardial infarction; H/R: hypoxia-reoxygenation; HCMs: human cardiac myocytes; HUVECs: human umbilical vein endothelial cells; ceRNA: competing endogenous RNA; HIF1A-AS1: hypoxia inducible factor 1*α*-antisense RNA 1; MALAT1: metastasis-associated lung adenocarcinoma transcript 1; TUG1: taurine-upregulated gene 1; APF: autophagy-promoting factor; GAS5: growth arrest specific 5; MEG3: maternally expressed gene 3; HOTAIR: HOX transcript antisense RNA; HULC: highly upregulated in liver cancer; IL-1*β*: interleukin-1*β*; UCA1: urothelial carcinoma-associated 1; NEAT1: nuclear paraspeckle assembly transcript 1; FTX: five prime to Xist; SNHG1: small nucleolar RNA host gene 1; RMRP: mitochondrial RNA-processing endoribonuclease; PINT: p53-induced transcript; NRF: necrosis-related factor; OIP5-AS1: Opa-interacting protein 5-antisense transcript 1; CARL: cardiac apoptosis-related lncRNA; MDRL: mitochondrial dynamic-related lncRNA; KCNQ1OT1: KCNQ1 opposite strand/antisense transcript 1; NLRC5: nucleotide-binding and oligomerization domain-like receptor C5; AKT: protein kinase B; PDCD4: programmed cell death 4; PTGS2: prostaglandin-endoperoxide synthase 2; NLRP3: nod-like receptor protein-3; BNIP3: Bcl2 19 kDa protein-interacting protein 3; SOCS2: suppressor of cytokine signaling 2; HDAC3: histone deacetylase 3; ATG7: autophagy-related gene 7; PI3K: phosphatidylinositol 3 kinase; FADD: Fas-associated protein with death domain; cIAP1: cellular inhibitor of apoptosis protein 1; PARP1: poly(ADP-ribose) polymerase 1; PPAR*α*: peroxisome proliferator-activated receptor *α*; Cab39: calcium-binding protein 39; MMP-2: matrix metalloproteinase-2; Bcl2: B-cell lymphoma-2; HSP70: heat shock protein 70; MAPK6: mitogen-activated protein kinase 6; ATG3/7/12: autophagy-related gene 3/7/12; Bax: B-cell lymphoma protein 2- (Bcl2-) associated X; PINK1: PTEN-induced putative kinase 1; Bcl2l2/12: B-cell lymphoma-2-like 2/12; HIF-1: hypoxia inducible factor-1; VEGF: vascular endothelial growth factor; Fmr1: fragile X mental retardation 1; FOXO4: forkhead box O4; CK: creatine kinase; CM-MB: creatine kinase MB form; LDH: lactate dehydrogenase; RIPK1/3: receptor-interacting serine/threonine-protein kinase 1/3; CSE: cystathionine-*γ*-lyase; SIRT1: sirtuin 1; AMPK: adenosine monophosphate-activated protein kinase; PGC1*α*: peroxisome proliferator-activated receptor-*γ* coactivator-1*α*; TRAF6: TNF receptor-associated factor 6; HMGB1: high-mobility group box 1; Rac1: Ras-related C3 botulinum toxin substrate 1; PHB2: prohibitin 2; LGALS3: galectin-3.

**Table 2:** The circRNA-miRNA-mRNA axis in I/R.

Tissues	Models	Species	Cell dysfunction	Expression	circRNA	miRNA	mRNA	Function	Mechanism	Relationship	Prediction tool	Ref
Myocardial	MI/R in mice and HUVECs under I/R	Human; mouse	Apoptosis; migration	↓	DLGAP4	miR-143	HECTD1	Had no effect on apoptosis in endothelial cells; attenuated the I/R-induced increase in endothelial cell migration	The negative regulation of mimic-miR-143 on HECTD1 protein was abolished by the overexpression of circDLGAP4	NR	Bioinformatics analysis	[61]
	IR	n/a	Apoptosis	↓	DLGAP4	miR-143	Bcl2	A potential regulated therapeutic target of cardiomyocyte apoptosis in myocardial I/R injury	Functions as an endogenous miR-143 sponge	n/a	n/a	[60]
	I/R in mice and H9C2 cells under H_2_O_2_ or H/R treatment	Mouse	Apoptosis	↑	NCX1	miR-133a-3p	CDIP1	Knockdown of circNCX1 in murine cardiomyocytes and heart tissues reduced the levels of CDIP1 and attenuated the apoptosis and I/R injury	Acting as an endogenous miR-133a-3p sponge	NR	RNA hybrid	[62]
	IR in mice and mice primary cardiomyocytes under A/R treatment	Mouse	Apoptosis	↑	MFACR	miR-652-3p	MTP18	The knockdown of MFACR attenuated the I/R-induced upregulation of mitochondrial fission, apoptosis, and MI size	Acts as a miR-652-3p sponge	NR	n/a	[63]
	HUVECs under H/R treatment	Human	Proliferation; migration; angiogenesis	↓	circ_100338	miR-200a-3p	FUS	Overexpression of circ_100338 promotes angiogenesis	circ_100338 can indeed regulate angiogenesis by binding to miRNA-200a-3p.	NR	n/a	[65]
	I/R in mice	Mouse	Apoptosis	↑	TLK1	miR-214	RIPK1	Overexpression of RIPK1 led to impaired cardiac function indexes, increased infarct area, and cell apoptosis	Acted as a sponge of miR-214	NR	starBase	[64]
Cerebral	NSCs under OGD/R condition	Mouse	Apoptosis	↑	cZNF292	miR-22	Wnt/b-catenin; PKC/ERK	Silencing cZNF292 alleviated OGD/R-stimulated damage in NSCs	miR-22 expression was negatively regulated by cZNF292	NR	n/a	[116]
	tMCAO/R in mice and mouse primary cortex neurons under OGD/R condition	Human; mouse	Apoptosis; atrophy	↑	TLK1	miR-335-3p	TIPARP	Knockdown of circTLK1 significantly decreased infarct volumes, attenuated neuronal injury, and improved neurological deficit	Functioned as an endogenous miR-335-3p sponge	NR	RNA hybrid; Arraystar, TargetScan; miRanda	[117]
	MCAO/R in mice	Mouse	Apoptosis	↑	circRNA_008018	miR-99a	PI3K/AKT/GSK3b	Knockdown of circ_008018 attenuated cerebral I/R-induced brain tissue damage and neurological deficits in mice	Inhibits the transcriptional activity of miR-99a	NR	starBase v.2.0; circBase	[118]
	tMCAO/R in mice and mouse primary astrocytes under OGD/R condition	Human; mouse	Autophagy	↑	Hectd1	miR-142	TIPARP	Knockdown of circHectd1 expression significantly decreased infarct areas, attenuated neuronal deficits, and ameliorated astrocyte activation in tMCAO mice	Functions as an endogenous miR-142 sponge	NR	RNA hybrid; TargetScan	[119]
	HT22 cells under OGD/R condition	Mouse	n/a	↑	circRNA_015947	miR-188-3p, miR-329-5p, miR-3057-3p, miR-5098, miR-683	99 target genes	Apoptosis-related pathways; metabolism-related pathways; immune-related pathways	May function as a sponge for its targeted miRNAs	n/a	TargetScan; miRanda	[115]
	HBMECs under OGD/R condition	Human	Proliferation; apoptosis; inflammation	↑	ANRIL	miR-622	p65 and I*κ*B*α*	Overexpression of circANRIL significantly inhibited the proliferation of OGD/R-induced HBMECs and aggravated OGD/R-induced cell apoptosis	Served as an miR-622 sponge	NR	Bioinformatics analysis	[120]
Hepatic	I/R in mice	Mouse	Inflammation	↑	mmu_circRNA_005186	miR-124-3p	Epha2	mmu_circRNA_005186 silencing attenuated the LPS-induced inflammation	Serving as a miRNA sponge for miR-124-3p	NR	Cytoscape software	[128]
Renal	HK-2 cells under I/R treatment	Human	Apoptosis; inflammation	↓	YAP1	miR-21-5p	PI3K/AKT/mTOR	CircYAP1 overexpression expedited cell growth and weakened secretion of inflammatory factors and ROS generation in I/R-disposed cells	Sponge to miR-21-5p	RC	Circular RNA Interactome	[140]
Intestinal	I/R in mice	Mouse	n/a	↓	circRNA_012412	miR-7649-3p	Sertad1	May play pivotal roles in endogenous protective signaling in iPoC	n/a	n/a	TargetScan; miRanda; miRDB	[149]
				↓	circRNA_012412	miR-3473c	Sertad1					
				↓	circRNA_012412	miR-6991-3p	Nudcd1					
				↓	circRNA_012412	miR-6991-3p	Jam2					

↑: the upward arrow indicates increased expression of circRNAs; ↓: the downward arrow indicates decreased expression of circRNAs; n/a: not applicable; NR: circRNAs negatively regulate miRNAs; PR: circRNAs positively regulate miRNAs; RC: reciprocal correlations between circRNAs and miRNAs; circRNA: circular RNA; miRNAs: microRNAs; IRI: ischemia-reperfusion injury; NSCs: neural stem cells; HUVECs: human umbilical vein endothelial cells; HBMECs: human brain microvascular endothelial cells; tMCAO/R: transient middle cerebral artery occlusion/reperfusion; OGD/R: oxygen-glucose deprivation and reperfusion; H/R: hypoxia-reoxygenation; A/R: anoxia/reoxygenation; NCX1: sodium/calcium exchanger 1; MFACR: mitochondrial fission and apoptosis-related circRNA; ANRIL: antisense noncoding RNA in the INK4A locus; YAP1: yes-associated protein 1; TLK1: serine/threonine-protein kinase tousled-like 1; Bcl2: B-cell lymphoma protein 2; HECTD1: HECT domain E3 ubiquitin protein ligase 1; CDIP1: cell death-inducing protein; MTP18: mitochondrial protein 18 kDa; RIPK1: receptor-interacting serine/threonine-protein kinase 1; Epha2: ephrin type-A receptor 2; PI3K: phosphatidylinositol 3-kinase; AKT: protein kinase B; mTOR: mammalian target of rapamycin; Sertad1: SERTA domain-containing protein 1; Nudcd1: NudC domain-containing protein 1; Jam2: junctional adhesion molecule B; PKC: protein kinase C; ERK: mitogen-activated protein kinase; TIPARP: TCDD inducible poly(ADP-ribose) polymerase; GSK3B: glycogen synthase kinase 3 beta.

**Table 3:** The lncRNA-miRNA-mRNA axis in cerebral I/R.

Models	Species	Cell dysfunction	Expression	lncRNA	miRNA	mRNA	Function	Mechanism	Relationship	Prediction tool	Ref
MCAO/R in mice and mice primary astrocytes under OGD/R condition	Mouse	Apoptosis	↑	MALAT1	miR-145	AQP4	Knockdown of MALAT1 increased cell viability and reduced cell apoptosis in MA-C cells	miR-145 was identified as a potential target of MALAT1	NR	starBase; TargetScan	[95]
Mouse primary BMECs under OGD/R condition	Mouse	Autophagy	↑	MALAT1	miR-26b	ULK2	MALAT1 promoted BMEC autophagy and survival under OGD/R condition	MALAT1 served as a ceRNA by sponging miR-26b	NR	lncRNA database v2.0; miRDB	[107]
MCAO/R in mice and mice primary cortical neurons under OGD/R condition	Mouse	Autophagy	↑	MALAT1	miR-30a	Beclin1	Downregulation of MALAT1 suppressed ischemic injury and autophagy in vitro and in vivo	May serve as a molecular sponge for miR-30a	NR	RNA hybird; starBase v.2.0	[108]
MCAO/R in mice and primary mouse astrocytes under OGD/R condition	Mouse	Apoptosis	↑	TUG1	miR-145	AQP4	Knockdown of TUG1 decreased lactate dehydrogenase levels and the ratio of apoptotic cells and promoted cell survival in vitro and reduced the infarction area and cell apoptosis in I/R mouse brains in vivo	May function as a ceRNA for miR-145	NR	Bioinformatic analysis	[96]
tMCAO/R in rats and PC-12 cells under OGD/R condition	Rat	Proliferation; apoptosis; inflammation	↑	H19	miR-138-5p	p65	H19 promotes inflammatory response and improved neurological function in tMCAO rat model	Negatively regulated miR-138-5p expression	NR	n/a	[97]
MCAO/R in mice and N2a cells under OGD/R condition	Mouse	Apoptosis	↑	H19	miR-148a-3p	Rock2	lncRNA-H19 altered OGD/R-induced oxidative stress	May act as a molecular sponge of miR-148a-3p	NR	starBase	[99]
MCAO/R in rats and neuronal cells under OGD/R condition	Human; rats	Apoptosis	↑	H19	miR-19a	Id2	Knockdown of H19 alleviated cell apoptosis, significantly decreased neurological deficit, brain infarct volume, and neuronal apoptosis	miR-19a directly binds to H19	NR	starBase 2.0	[98]
MCAO/R in mice and N2a cells under OGD/R condition	Mouse	Apoptosis	↓	Rian	miR-144-3p	GATA3	Overexpression of Rian could inhibit the cell apoptosis induced by OGD and distinctly reduce the infarct size	miR-144-3p directly interacts with Rian	NR	DIANA online tools	[100]
MCAO/R in rats and SK-N-SH and SH-SY5Y cells under OGD/R condition	Rat	Pyroptosis	↑	MEG3	miR-485	AIM2	Knockdown of MEG3 inhibited OGD/R-induced pyroptosis and inflammation, and inhibited Caspase-1 signaling and decreased the expression of AIM2, ASC, cleaved-Caspase-1 and GSDMD-N	Acted as a molecular sponge to suppress miR-485	NR	n/a	[114]
MCAO/R in mice and N2a cells under OGD/R condition	Mouse	Apoptosis	↑	MEG3	miR-21	PDCD4	Knockdown of MEG3 protects against ischemic damage and improves overall neurological functions in vivo	Functions as a ceRNA for directly binding to miR-21	NR	lnCeDB	[144]
N2a cells under OGD/R condition	Mouse	Apoptosis	↑	Gm11974	miR-766-3p	NR3C2	Knockdown of lncRNA Gm11974 alleviated the apoptosis induced by OGD and cell death rates were significantly reduced	Could negatively regulate the expression of miR-766-3p	NR	Bioinformatics software	[101]
MCAO/R in mice and N2a cells under OGD/R condition	Mouse	Apoptosis	↑	Oprm1	miR-155	GATA3	Overexpression of lncRNA Oprm1 alleviated the apoptosis induced by OGD and distinctly decreased infarct size	miR-155 is a direct target of lncRNA Oprm1	NR	TargetScan	[102]
tMCAO/R in mice and N2a cells under OGD/R condition	Human; mouse	Autophagy	↑	KCNQ1OT1	miR-200a	FOXO3/ATG7	Knockdown of KCNQ1OT1 remarkably reduced the infarct volume and neurological impairments in tMCAO mice and might inhibit I/R-induced autophagy and increase cell viability	Acted as a ceRNA of miR-200a	NR	Lncbase	[109]
Mice primary cortical neurons under OGD/R condition	Mouse	Apoptosis	↑	KCNQ1OT1	miR-9	MMP8	KCNQ1OT1 regulates OGD/R-induced injury in cultured primary cortical neurons via modulating miR-9/MMP8 axis as a ceRNA	Directly binds to miR-9	NR	Bioinformatic analysis	[106]
MCAO/R in mice and N2a cells under OGD/R condition	Mouse	Apoptosis	↑	AK038897	miR-26a-5p	DAPK1	AK038897 knockdown protected against MCAO/R-induced brain injury and neurological deficits in vivo	Functions as a ceRNA for miR-26a-5p	NR	starBase; TargetScan	[103]
MCAO/R in mice and N2a cells under OGD/R condition	Mouse	Apoptosis	↑	CHRF	miR-126	SOX6	CHRF knockdown in vivo markedly prevented ischemic damage and alleviated neurological dysfunctions	Played as a ceRNA to direct binding with miR-126	NR	TargetScan	[104]
SH-SY5Y cells under OGD/R condition	Human	Apoptosis	↓	SNHG16	miR-106b-5p	LIMK1	lncRNA SNHG16 promoted OGD/R-induced SH-SY5Y cell survival and suppressed its apoptosis and Caspase-3 activity	Directly targeted miR-106b-5p	NR	starBase; MiRTarBase	[105]
MCAO/R in rats and PC-12 cells under OGD/R condition	Rat	Inflammation	↑	SNHG14	miR-136-5p	ROCK1	Interference of SNHG14 by shRNA vector enhanced neuron survival and suppressed inflammation in response to OGD/R insult. SNHG14 promoted neurological impairment and inflammatory response	Acting as a sponge of miR-136–5p	NR	starBase	[113]
N2a cells under OGD/R condition	Mouse	Proliferation; apoptosis	↑	SNHG12	miR-199a	SIRT1	Knockdown SNHG12 inhibited cell proliferation under OGD/R condition	SNHG12 blocks the expression of miR-199a	NR	http://microRNA.org	[110]
Mouse primary BMECs under OGD/R condition	Mouse	Cell death; inflammation	↑	SNHG12	miR-199a	n/a	Overexpression of SNHG12 inhibited BMEC death and the inflammatory response but promoted angiogenesis after OGD/R	Directly targets miR-199a	NR	miRcode	[111]
Mouse primary BMECs under OGD/R condition	Mouse	Cell death; migration	↑	SNHG1	miR-199a	HIF-1*α*; VEGF	Promoted BMEC survival under OGD/R condition, and angiogenesis after OGD/R treatment	Snhg1 targets miR-199a by binding to complementary miR-199a seed regions	NR	miRDB	[112]

↑: the upward arrow indicates increased expression of lncRNAs; ↓: the downward arrow indicates decreased expression of lncRNAs; n/a: not applicable; NR: lncRNAs negatively regulate miRNAs; PR: lncRNAs positively regulate miRNAs; RC: reciprocal correlations between lncRNAs and miRNAs; tMCAO/R: transient middle cerebral artery occlusion/reperfusion; OGD/R: oxygen-glucose deprivation and reperfusion; BMECs: brain microvascular endothelial cells; ceRNA: competing endogenous RNA; Rian: RNA imprinted and accumulated in nucleus; MALAT1: metastasis-associated lung adenocarcinoma transcript 1; TUG1: taurine-upregulated gene 1; MEG3: maternally expressed gene 3; CHRF: cardiac hypertrophy-related factor; HOTAIR: HOX transcript antisense RNA; SNHG1/12/14/16: small nucleolar RNA host gene 1/12/14/16; LIMK1: the LIM motif-containing protein kinase family-contained LIM kinase 1; KCNQ1 opposite strand/antisense transcript 1; MMP-8: matrix metalloproteinase-8; Id2: inhibitor of DNA binding/differentiation 2; GATA3: GATA-binding protein 3; ULK2: Unc-51-like kinase 2; AQP4: aquaporin 4; Rock2: Rho-associated protein kinase 2; AIM2: absent in melanoma 2; SOX6: sex-determining region Y box 6; HIF-1*α*: hypoxia inducible factor-1*α*; VEGF: vascular endothelial growth factor; SIRT1: sirtuin 1; GATA3: GATA-binding protein 3; ROCK1: Rho-associated coiled-coil-containing protein kinase 1; FOXO3: forkhead box O3; ATG7: autophagy-related gene 7; DAPK1: death-associated protein kinase 1; NR3C2: nuclear receptor subfamily 3 group C member 2.

**Table 4:** The lncRNA-miRNA-mRNA axis in other organs I/R.

Tissues	Models	Species	Cell dysfunction	Expression	lncRNA	miRNA	mRNA	Function	Mechanism	Relationship	Prediction tool	Ref
Hepatic	I/R in rats and BRL-3A cells under H/R treatment	Rats	Apoptosis	↑	Gm4419	miR-455	SOX6	Knockdown of Gm4419 alleviated I/R-induced liver damage and alleviated H/R-induced apoptosis	Could sponge miR-455	NR	miRDB; TargetScan	[122]
	IRI in mice and mice primary hepatocytes under H_2_O_2_ treatment	Mouse	Autophagy	↑	HOTAIR	miR-20b-5p	ATG7	Knockdown of the expression of HOTAIR attenuated autophagy induced by hydrogen peroxide	Function as ceRNA for miR-20b-5p	NR	TargetScan; starBase	[127]
	HI/R in mice and HL7702 under H/R treatment	Mouse	Apoptosis	↓	MEG3	miR-34a	Nrf2	MEG3 overexpression could improve hepatic function of HIR mice, and markedly decreased the expression of serum ALT and AST	Functioned as a ceRNA for miR-34a	NR	DIANA tools	[123]
	IRI in mice and BNL-CL2 cells under H/R treatment	Mouse	Apoptosis	↑	AK054386	miR-199	CHOP	Increased expression of AK054386, which might be mediated by activated NF-*κ*B, resulted in sustained ERS and increased cell apoptosis and death in hepatic IRI mouse and cellular models	Acted as a ceRNA of miR-199	NR	RNA22	[124]
Renal	I/R in mice and HK-2 cells under H/R treatment	Human; mouse	Apoptosis	↑	GAS5	miR-21	TSP-1	GAS5 facilitated apoptosis in renal I/R injury	Competitively sponging miR-21	RC	n/a	[134]
	IRI in rats and HK-2 cells under OGD/R treatment	Rats	Inflammation; apoptosis	↑	TUG1	miR-449b-5p	HMGB1; MMP2	TUG1 silencing attenuates I/R-induced inflammation and apoptosis	miR-449b-5p was a direct target of TUG1	NR	starBase	[137]
	HK-2 cells under H/R treatment	Human; rat	Apoptosis	↑	NEAT1	miR-27a-3p	n/a	Repression the expression of NEAT1 decreased CoCl2-induced injury in HK-2	NEAT1 was a direct target of miR-27a-3p and miR-27a-3p was a direct target of NEAT1	RC	TargetScan; starBase; http://microRNA.org	[136]
	IRI in mice	Mouse	Proliferation	↑	MALAT1	miR-139-5p	SPRR2F, SPRR1A, MMP-10	Noncoding RNAs MALAT1 and miR-139-5p were involved in IRI	n/a	NR	n/a	[133]
	IRI in rats and HK-2 cells under H/R treatment	Human; rats	Apoptosis	↑	LINC00520	miR-27b-3p	OSMR	Knockdown of LINC00520 reduced acute renal injury both in vitro and in vivo	LINC00520 binds to miR-27b	NR	Cytoscape software	[139]
	AKI in rats and HK-2 cells under CoCl2 treatment	Human; rats	Proliferation; apoptosis	↑	XIST	miR-142-5p	PDCD4	Knockdown of PDCD4 rescued the effects of CoCl2 on the proliferation and apoptosis of HK-2 cells	miR-142-5p was a target of XIST	NR	Bioinformatic analysis	[138]
Testicular	IRI in mice and GC-1 spermatogenic cells under OGD/R treatment	Mouse	Apoptosis; proliferation	↑	MALAT1	miR-214	TRPV4	MALAT1 promotes cell apoptosis and suppresses cell proliferation in vitro and in vivo	Directly binds to miR-214	NR	LncBase experimental v.2 database	[145]
Intestinal	IR in mice	Mouse	Proliferation	n/a	H19	miR-675	ZO-1/E-cadherin	H19 overexpression resulting in the dysfunction of the epithelial barrier	Serving as a precursor for miR-675	PR	n/a	[148]
Retinal	IRI in mice and newborn mouse primary RGCs under ischemic treatment	Mouse	Apoptosis	↑	Mbd2-AL1	miR-188-3p	Traf3	Mbd2-AL1 mediates I/R-induced RGC apoptosis	Sponged miR-188-3p	NR	RegRNA	[142]
	I/R in mice and neonatal mouse primary retinal microglia and RGCs under OGD/R treatment	Mouse	Pyroptosis; apoptosis; inflammation	↑	H19	miR-21	PDCD4	Increased lncRNA-H19 expression significantly promotes NLRP3/6 inflammasome imbalance and results in microglial pyroptosis, cytokine overproduction, and neuronal death	Functioned via sponging miR-21	NR	n/a	[143]
Spinal cord	I/R in rats and SH5Y-SY cells under OGD/R treatment	Rat	Apoptosis	↓	CasC7	miR-30c	Beclin1	Knockdown of CasC7 could promote cell apoptosis and downregulate miR-30c target gene expression	Functioned as a miR-30c decoy	NR	n/a	[146]
	IRI in rats and HN neuronal cells under OGD/R treatment	Rat	Apoptosis	↓	MALAT1	miR-204	Bcl2	Knockdown of MALAT1 increased cell apoptosis	Overexpression of MALAT1 downregulated miR-204	NR	n/a	[147]

↑: the upward arrow indicates increased expression of lncRNAs; ↓: the downward arrow indicates decreased expression of lncRNAs; n/a: not applicable; NR: lncRNAs negatively regulate miRNAs; PR: lncRNAs positively regulate miRNAs; RC: reciprocal correlations between lncRNAs and miRNAs; IRI: ischemia-reperfusion injury; AKI: acute kidney injury; H/R: hypoxia-reoxygenation; ceRNA: competing endogenous RNA; MALAT1: metastasis-associated lung adenocarcinoma transcript 1; TUG1: taurine-upregulated gene 1; Mbd2-AL1: methyl-CpG-binding domain protein 2-associated long noncoding RNA 1; GAS5: growth arrest specific 5; MEG3: maternally expressed gene 3; HOTAIR: HOX transcript antisense RNA; NEAT1: nuclear paraspeckle assembly transcript 1; CasC7: cancer susceptibility candidate 7; TRPV4: transient receptor potential vanilloid 4; SPRR2F: small proline-rich protein 2F; SPRR1A: small proline-rich protein 1A; MMP-2/10: matrix metalloproteinase-2/10; CHOP: C/EBP homologous protein; HMGB1: high-mobility group box 1; Bcl2: B-cell lymphoma-2; Traf3: tumor necrosis factor (TNF) receptor-associated factor 3; XIST: X chromosome inactivation; PDCD4: programmed cell death 4; TSP-1: thrombospondin 1; ZO-1: zonula occludens 1; Nrf2: nuclear factor erythroid 2-related factor; ATG7: autophagy-related gene 7; Bax: B-cell lymphoma protein 2- (Bcl2-) associated X; Bcl2: B-cell lymphoma protein 2; SOX6: sex-determining region Y box 6; OSMR: oncostatin M receptor *β*.
